# 

**Published:** 1862-04

**Authors:** 


### CONTENTS.---APRIL, 1862.

### ORIGINAL CONTRIBUTIONS.

PAGE

ART. IX. Ovarian Tumors. By Wm. H. Byford, A. M., M. D., Professor of Obstetrics and Diseases of Women and Children, in the Medical Department, Lind University, Chicago, Ill.,

ART. XI. Valedictory Address, by J. H. Hollister, M. D., Professor of Anatomy,.... 215

ART. XII. A Fatal Case of a Foreign Body in the Air Passages. By John Bartlett, M. D., Pesotum, Ills.,.................. .................................................. 229

Annual Meeting of the Chicago Medical Society,.................................... 232

### SELECTIONS.

Clinical Lecture on Anæmia. By Thos. K. Chambers, M. D.,......................... 235

Rules for Guidance in Chloroform Accidents. By Dr. Kidd,............................ 242

### BOOK NOTICES.

Anatomy, Descriptive and Surgical. By Henry Gray, F. R. S., etc., .... ............ 244

### EDITORIAL.

Postponement of the Annual Meeting of the Illinois Medical Society,..................... 246

Metropolitan Health Bill of New York,.................................................. 247

Reserve Corps of Army Surgeons,....................................................... 248

Personal—Dr. Andrews,........ .................... ................................ 249

The London Lancet,.................................................................... 249

Obituary—E. B. Rockwell, M. D., ...................................................... 249

Paralysis,............................................................................ 250

Aconite and Nux Vomica Antidotal to each other,......................................... 254

Asthma--The Blane Medals—Desire for Information in Spain--Physicians and Surgeons,. 255

